# Lewis fucose is a key moiety for the recognition of histo‐blood group antigens by GI.9 norovirus, as revealed by structural analysis

**DOI:** 10.1002/2211-5463.13370

**Published:** 2022-01-30

**Authors:** Tomomi Kimura‐Someya, Miyuki Kato‐Murayama, Kazushige Katsura, Naoki Sakai, Kazutaka Murayama, Kazuharu Hanada, Mikako Shirouzu, Yuichi Someya

**Affiliations:** ^1^ 208578 RIKEN Center for Biosystems Dynamics Research Yokohama Japan; ^2^ Graduate School of Biomedical Engineering Tohoku University Sendai Japan; ^3^ Department of Virology II National Institute of Infectious Diseases Tokyo Japan; ^4^ Present address: Department of Biochemistry and Cell Biology National Institute of Infectious Diseases Tokyo Japan

**Keywords:** crystal structure, fucose, histo‐blood group antigens, Lewis antigens, norovirus, VP1 protein

## Abstract

Noroviruses have been identified as major causative agents of acute nonbacterial gastroenteritis in humans. Histo‐blood group antigens (HBGAs) are thought to play a major role among the host cellular factors influencing norovirus infection. Genogroup I, genotype 9 (GI.9) is the most recently identified genotype within genogroup I, whose representative strain is the Vancouver 730 norovirus. However, the molecular interactions between host antigens and the GI.9 capsid protein have not been investigated in detail. In this study, we demonstrate that the GI.9 norovirus preferentially binds Lewis antigens over blood group A, B, and H antigens, as revealed by an HBGA binding assay using virus‐like particles. We determined the crystal structures of the protruding domain of the GI.9 capsid protein in the presence or absence of Lewis antigens. Our analysis demonstrated that Lewis fucose (α1–3/4 fucose) represents a key moiety for the GI.9 protein–HBGA interaction, thus suggesting that Lewis antigens might play a critical role during norovirus infection. In addition to previously reported findings, our observations may support the future design of antiviral agents and vaccines against noroviruses.

## Graphical abstract

Here we demonstrated the preferential binding of the GI.9 norovirus to Lewis antigens over blood group A, B, and H antigens . In addition, X‐ray crystallography revealed that the VP1 capsid protein interacted mainly with the fucose moiety (α1–3/4‐Fuc, ‘LeFuc’) of Lewis antigens, thus suggesting a critical role of Lewis antigens during norovirus infection. Our observations could support the future design of antiviral agents and vaccines against noroviruses.

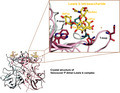

**Abbreviations**
ABH: blood group A, B and H antigens
D‐PBS: Dulbecco's modified PBS
FUT: fucosyltransferase
GI: genogroup I
GII: genogroup II
HBGA: histo‐blood group antigen
HRP: horseradish peroxidase
HSA: human serum albumin
ORF: open‐reading frame
SUMO: small ubiquitin‐related modifier
VLP: virus‐like particle
VP: viral protein

Noroviruses are leading causative agents of nonbacterial acute gastroenteritis in humans and are classified into the family *Caliciviridae* [1]. Among the 10 genogroups, genogroup I (GI) and genogroup II (GII) noroviruses are major pathogens for humans and are further classified into nine and 27 genotypes, respectively [2]. More than 90% of human noroviruses isolated from patients belong to GII [3]. GII, genotype 4 (GII.4) noroviruses have been always dominant, sometimes with minor modification that are recognized as subtypes or variants. Emerging genotypes in both GI and GII might pose a threat to humans, since considerable numbers of humans, especially infants and young children, are thought not to have an effective immunity against them.

The GI.9 norovirus is a newly identified genotype, the representative strain of which is the Vancouver 730 strain isolated in Canada in 2004. Since then, several GI.9 isolates have been discovered in several countries. To determine the reasons for the emergence of new genotypes, their evolutionary implications, and the best means of controlling infection by these genotypes, it will be important to clarify and compare the structures of noroviruses from various genotypes.

A single norovirus virion is composed of 180 molecules of the viral protein 1 (VP1), a major capsid protein, which is encoded by open‐reading frame 2 (ORF2) in the positive sense, single‐stranded RNA genome, and 90 dimers of VP1 proteins self‐assemble to form a *T* = 3 icosahedral particle [4]. ORF3 encodes a minor structural protein abundant in basic amino acids, the VP2 protein. It is therefore suggested that the VP2 protein interacts with the RNA genome. It was shown that the VP2 protein of feline calicivirus formed a portal‐like assembly following its receptor binding, which possibly functioned as a genome‐translocating channel [5]. Since feline caliciviruses also belong to the family *Caliciviridae* and are closely related to noroviruses, the VP2 proteins from noroviruses are presumed to have the same function. When the norovirus *ORF2* even without the *ORF3* is expressed in insect cells via recombinant baculoviruses, virus‐like particles (VLPs) without the genome are formed and excreted into the culture medium [6]. Normally, VLPs of 38 nm in diameter are formed, but sometimes VLPs of 23 nm in diameter are produced [7, 8]. These smaller particles have a *T* = 1 icosahedral symmetry [9].

The crystal structure of the 38‐nm VLPs from the GI.1 Norwalk strain was solved in 1999 [4]. The structure showed that the VP1 protein consisted of two domains, an S domain that forms a contiguous spherical shell and a P domain that protrudes from the shell. The P domain is further divided into two subdomains, P1 and P2. Compared with those in the P1 subdomain, amino acids in the P2 subdomain are less conserved, which is attributed to the presence of a wide variety of genotypes and hence the differences in antigenicity. The P2 subdomain is inserted into the P1 subdomain and resides on the outmost surface of the virus capsid [4].

The P domain proteins self‐assemble into dimers (P dimers) when expressed in *Escherichia coli* cells. P dimers derived from various GI norovirus strains have been subjected to crystal structure determination [10, 11, 12, 13, 14]. Also, in the case of GII noroviruses, the high‐resolution structures revealed the molecular basis for the interaction between the P dimers and histo‐blood group antigens (HBGAs), indicating the difference in the HBGA‐binding mode between GI and GII noroviruses [15, 16, 17, 18, 19, 20, 21, 22]. It is noteworthy that norovirus binds to synthetic HBGAs having the same saccharide structures as those found on the cell surface or in secretions. Generally, nonsecretors with the null fucosyltransferase (FUT)2 allele are tolerant to norovirus infection [23], but it is known that a few genotypes are capable of developing the norovirus illness even in nonsecretors [24], suggesting that Lewis antigens might be involved in norovirus infection. These observations raise the possibility that some HBGAs—namely, blood group A, B and H (ABH) antigens and/or Lewis antigens—are receptors for noroviruses, but their physiological roles in norovirus infection remain unknown. Interestingly, the HBGA binding profile varies among genotypes even within the same genogroup.

In this study we determined the crystal structures of P dimers from the emerging GI.9 norovirus in the absence or presence of Lewis antigens to assess the structural basis for the HBGA recognition by this genotype.

## Materials and methods

### Norovirus strain and plasmid construction

A gene fragment encoding the VP1 and VP2 proteins of the Vancouver strain (GenBank ID: HQ637267) was synthesized by GenScript (Piscataway, NJ, USA). Codons for this fragment were optimized for expression in insect cells. The entire VP1 and VP2 region was inserted into a pORB baculovirus transfer vector (Allele Biotechnology, San Diego, CA, USA), resulting in the construction of pORB Vancouver‐VP1,2.

For the expression of P domain proteins from the Vancouver VP1, the gene fragment encoding the P domain, including the amino acids from positions 229 to 540, was attached by overlap PCR to sequences encoding a modified poly‐histidine (N11, MKDHLIHNHHKHEHAHAEH) affinity tag [25] and a small ubiquitin‐related modifier (SUMO) fusion tag at the N terminus, and then subcloned into the plasmid pCR2.1‐TOPO (Thermo Fischer Scientific, Waltham, MA, USA). The resultant plasmid expressed the N‐terminal SUMO fusion protein of the P domain.

### Preparation of VLPs

Sf9 cells (Oxford Expression Technologies, Oxford, UK) were transfected with pORB Vancouver‐VP1,2 together with Sapphire Baculovirus DNA (Allele Biotechnology), which resulted in the production of recombinant baculoviruses that could be used for VLP production in *Trichoplusia* *ni* cells (Oxford Expression Technologies). VLPs collected from culture media were separated by isopycnic CsCl density gradient centrifugation. A band including VLPs was diluted in distilled water and subjected to ultracentrifugation. Sedimented VLPs were resuspended in distilled water and stored at 4 °C. The integrity of VLPs was evaluated by transmission electron microscopy with uranyl acetate used as a stain.

### Preparation of P domain proteins

The N11‐tagged SUMO fusions of the Vancouver P domain proteins were synthesized by an *E. coli* cell‐free protein synthesis system [26] or expressed in the *E. coli* KRX strain by induction with 0.1% rhamnose. The fusion proteins were immobilized by a HisTrap column (GE Healthcare, Piscataway, NJ, USA), washed with 20 mm imidazole, and then eluted with 200 mm imidazole or a linear gradient of 20–250 mm imidazole. Fusion proteins were digested by a SUMO protease, followed by 2^nd^ HisTrap chromatography. The resultant flow‐through fraction containing cleaved P domain proteins without N11‐SUMO was desalted by using a HiPrep Desalting column (GE Healthcare) and then subjected to anion exchange chromatography using a HiTrapQ column (GE Healthcare). The P domain proteins were eluted by applying an NaCl gradient. The peak fraction was further applied to a HiLoad200 column (GE Healthcare) to purify to homogeneity. The purified P domain proteins were concentrated by using an Amicon Ultra 4 (MWCO 10,000; Merck Millipore, Burlington, MA, USA) to a protein concentration of around 10 mg·mL^−1^ in 10 mm Tris/HCl (pH 8.0).

### HBGA binding assay

The BSA conjugates of blood group A trisaccharide, blood group B trisaccharide, blood group H type 1 (lacto‐*N*‐fucopentaose I), Lewis a (lacto‐*N*‐fucopentaose II), Lewis x (lacto‐*N*‐fucopentaose III), and Lewis b (lacto‐*N*‐difucohexaose I) were purchased from Dextra Laboratories (Reading, UK). The HSA conjugates of Lewis y tetrasaccharide and lactose were purchased from IsoSep (Tullinge, Sweden). The lactose‐BSA conjugate was obtained from Sigma (St. Louis, MO, USA). BSA and HSA were purchased from FUJIFILM Wako Pure Chemical Corporation (Osaka, Japan) and Nacalai Tesque (Kyoto, Japan), respectively. BSA, HSA, and the BSA or HSA conjugates of lactose were used as negative controls in the HBGA binding assay. BSA, HSA, and the HBGA‐conjugates were solved in phosphate buffer (0.125 m KH_2_PO_4_, 0.425 m Na_2_HPO_4_, pH 6.8) at 1 mg·mL^−1^ and stored at −80 °C. BSA, HSA, and the conjugates were diluted in 50 mm sodium carbonate buffer (pH 9.6) at 20 µg·mL^−1^, and a 96‐well ELISA microplate was coated by using 50 µL·well^−1^ of the diluents at 4 °C overnight. After washing with Dulbecco's modified PBS (D‐PBS) containing 0.05% Tween 20, the microplate was blocked by dilution buffer (D‐PBS containing 0.5% Tween 20 and 5% skim milk) for 1 h at 37 °C. After washing, 50 µL of the dilution buffer was poured into each well. Fifty microliters of VLP suspension in dilution buffer at 100 µg·mL^−1^ was added to the wells in line B of the microplate, and twofold serial dilutions of VLPs were made to line G, followed by incubation at 37 °C for 1 h. After washing, rabbit antiserum raised against Vancouver VLPs (Scrum, Tokyo, Japan) diluted in dilution buffer at 1:4000 was added to each well, followed by incubation at 37 °C for 1 h. Then horseradish peroxidase (HRP)‐conjugated donkey antirabbit IgG (Abcam, Cambridge, MA, USA) was added and the microplate was placed at 37 °C for 1 h. 2,2′‐azino‐bis(3‐ethylbenzothiazoline‐6‐sulphonic acid) (Roche, Basel, Switzerland) was used as the HRP substrate, and the absorbance at 405 nm was measured; the absorbance at 630 nm was simultaneously recorded as a reference. For data analysis with the kaleidagraph software (Hulinks, Tokyo, Japan), the absorbance at 630 nm was subtracted from the absorbance at 405 nm.

### X‐ray crystallography of P domain proteins

The Vancouver P domain proteins were crystalized by sitting drop vapor diffusion at 20 or 25 °C with 0.1 m bicine (pH 9.0) containing 1.5–1.7 m MgCl_2_ as a reservoir solution. To obtain crystals of P domain protein complexes with Lewis oligosaccharides, the P dimer crystals were soaked with 100 mm Lewis b tetrasaccharide or Lewis x trisaccharide (FUJIFILM Wako) solution solved in the reservoir solution at 25 °C overnight. X‐ray diffraction data from the above preparations were collected at BL26B2 of SPring‐8 [27, 28, 29, 30] or by an in‐house X‐ray diffractometer (Rigaku FR‐E) (Rigaku Corporation, Tokyo, Japan). The diffraction data were processed with the XDS [31] or the HKL‐2000 [32] programs, and the structure was solved by molecular replacement, using the Phaser program [33] in the Phenix suite [34], using the P domain of the GI.8 Boxer strain (PDB ID: 4RDJ) for the apo‐form, or the P domain of the GI.9 Vancouver strain solved in this study for Lewis b‐ and Lewis x‐bound forms, respectively, as the search model. The refinement was conducted using the Phenix programs, and the structure was manually rebuilt with the Coot program [35]. Data collection and refinement statistics are presented in Table 1. The waals software (Altif Laboratories, Tokyo, Japan) and UCSF Chimera [36] were also used for structure visualization.

**Table 1:** Data collection and refinement statistics.

	Apo	Complex with Lewis b	Complex with Lewis x
Data collection			
Beamline (diffractometer)	SPring‐8, BL26B2	(RIGAKU FR‐E)	(RIGAKU FR‐E)
Space group	*C*2	*C*2	*C*2
Cell dimensions			
*a*, *b*, *c* (Å)	170.95 103.55 76.91	183.75, 76.46, 103.28	184.20, 77.24, 103.53
α, β, γ (°)	90.00 116.66 90.00	90.00, 124.20, 90.00	90.00, 124.20, 90.00
Resolution range^a^	48.0–2.10 (2.18–2.10)	50.0–2.40 (2.49–2.40)	50.0–2.26 (2.34–2.26)
Total reflections	263 175	170 366	209 926
Unique reflections	69 314	45 674	56 256
Completeness (%)^a^	99.5 (98.1)	98.2 (91.6)	99.6 (98.6)
Redundancy^a^	3.8 (3.7)	3.7 (3.3)	3.7 (3.5)
*I*/σ (*I*)^a^	6.3 (1.1)	18.9 (3.4)	15.3 (3.8)
*R*_meas_ ^a^	0.248 (2.063)	0.086 (0.395)	0.103 (0.411)
Refinement			
Resolution (Å)	2.10	2.40	2.26
*R*_work_	0.237	0.165	0.165
*R*_free_	0.269	0.196	0.195
RMS deviations			
Bond lengths (Å)	0.008	0.008	0.008
Bond angles (°)	0.960	0.973	0.931
Average *B* factors (Å ^2^)			
Overall	46.1	37.9	36.5
Macromolecules	46.1	37.8	36.0
Ligands	51.3	41.7	43.7
Solvent	45.8	38.7	39.7
Ramachandran plot (%)			
Favored	94.64	96.27	96.75
Allowed	5.36	3.73	3.25
Outliers	0.00	0.00	0.00
PDB ID	7VP0	7VS8	7VS9

^a^ Numbers in parentheses refer to the highest resolution shell.

## Results

### HBGA binding profile of the GI.9 Vancouver VLP

**Fig. 1:**
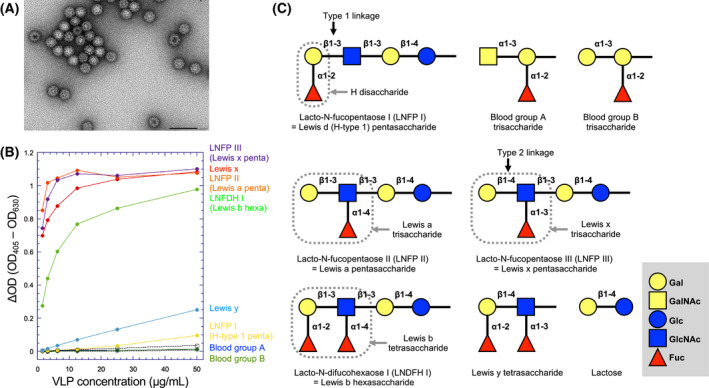
HBGA binding profile of the GI.9 Vancouver VLP. (A) Electron microscopic image of VLPs prepared from a *T*. *ni* insect cell culture. The bar indicates 100 nm. (B) ELISA‐based HBGA binding assay using VLPs. The assay was performed (*n* = 2) as described in the Materials and methods. The commercially available conjugates of the ABH and Lewis antigens with BSA or HSA were used, and are represented by different colors with the name of the HBGA. The lactose‐BSA (open circles with a dotted line) and lactose‐HSA (open squares with a dotted line) conjugates were used as negative controls. Each of the neolacto‐series BSA conjugates included the following oligosaccharides: LNFP I, Blood group H type 1 pentasaccharide (yellow); LNFP II, Lewis a pentasaccharide (orange); LNFP III, Lewis x pentasaccharide (purple); and LNDFH I, Lewis b hexasaccharide (yellow green). Blood groups A (blue) and B (green), and Lewis x (red) conjugates include the respective terminal trisaccharides, and the Lewis y conjugate (sky blue) includes the terminal tetrasaccharide. (C) Symbolic representation of the HBGAs used for the binding assay. The structures of HBGAs used for the binding assay are depicted in the conventional manner.

To determine the HBGA binding profile of the GI.9 Vancouver VLPs, an ELISA‐based binding assay was performed using commercially available BSA or HSA conjugates of HBGA. As shown in Fig. 1, the Vancouver VLPs bind to four types of Lewis antigens with high avidity. Lewis a and x antigens are known as nonsecretor antigens, which are predominantly produced in secretions of FUT2‐deficient nonsecretor individuals. Moreover, the VLPs bind to the secretor antigens Lewis b and Lewis y. The avidity for Lewis b is comparable to those for Lewis a and Lewis x, while the binding to Lewis y is relatively weak but notable. In contrast, the Vancouver VLPs bind to neither of the ABH antigens, although there was a weak signal of binding to the H antigen when a large amount of VLPs was used.

### Crystal structure of the GI.9 Vancouver P dimer

**Fig. 2:**
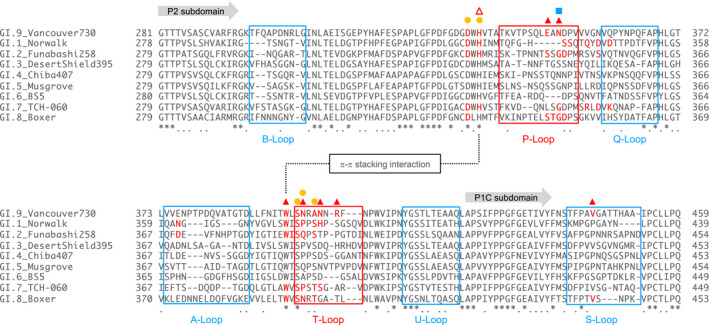
Amino acid alignment of VP1 proteins from the GI noroviruses. Amino acid sequences of the P2 subdomain and part of the C‐terminal P1 (‘P1C’) subdomain were aligned with the help of genetyx‐mac software (GENETYX Corp., Tokyo, Japan). Asterisks (*) indicate positions at which amino acid residues are conserved, and periods (.) indicate positions occupied by similar amino acids. Amino acid residues responsible for HBGA binding are shown with red letters. For information on the structures of the GI.1, GI.2, GI.7, and GI.8 P domains, published reports [10, 11, 12, 13, 14] were referenced. The roles of each residue of the GI.9 Vancouver VP1 are represented with symbols as follows: red closed triangles for LeFuc (α1–3/4 Fuc) binding; orange circles for Gal binding; red open triangles for SeFuc (α1–2 Fuc); blue squares for GlcNAc binding. Loop regions are boxed with the respective name.

To gain insight into the structural basis for the Lewis antigen binding to the GI.9 VP1 protein, the P domain proteins were synthesized. The size‐exclusion chromatogram of synthesized proteins shows that most of the P domain proteins form dimers (data not shown). The crystal structure of the dimerized Vancouver P domains, the P dimer, in apo‐form was solved at 2.10 Å. The overall structure of the P dimer (Fig. S1) was similar to those from other GI genotypes [10, 11, 12, 13, 14]—that is, GI.1, GI.2, GI.7, and GI.8, with RMSDs of 2.587, 2.578, 2.090, and 1.590 Å, respectively. Among the GI noroviruses, the GI.9 Vancouver strain has the longest B‐loop, P‐loop, and A‐loop and the shortest T‐loop, while the S‐loop is relatively long (Fig. 2). Consistent with their lengths, the B‐loop and A‐loop protrude outward to a greater extent than their counterparts from the GI.1 Norwalk strain, which are the shortest among the GI strains (Fig. S1B).

### Crystal structures of P dimer complexes with Lewis antigens

**Fig. 3:**
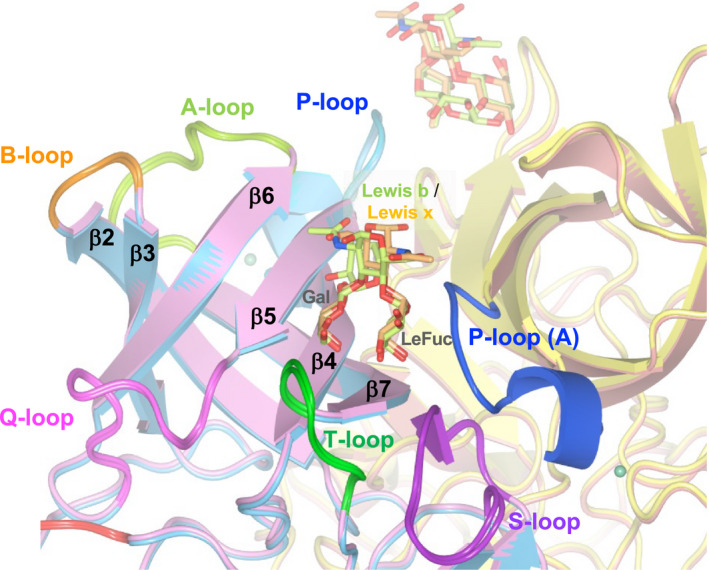
HBGA binding site in the GI.9 Vancouver P dimer. P dimers were purified and crystalized as described in the Materials and methods. The P dimer crystals were soaked with Lewis b tetrasaccharide or Lewis x trisaccharide to obtain the respective complex. The structures of the Lewis b complex and the Lewis x complex are overlaid. This figure focuses on the HBGA binding site on the B subunit of the P dimer. The P‐loop from the A subunit (P‐loop (A)) is involved in making this site. The loop structures and their names are depicted in the same color. Lewis b tetrasaccharide and Lewis x trisaccharide are depicted in yellow green and pale orange, respectively. The backbones of the A and B subunits of the Lewis b complex are depicted in pale red and sky blue, respectively, and those of the A and B subunits of the Lewis x complex are shown in yellow and pink, respectively.

To reveal the degree of difference between the binding modes of secretor Lewis antigens and those of nonsecretor Lewis antigens, we obtained the crystal structures of the P domains complexed with each of Lewis b tetrasaccharide and Lewis x trisaccharide (Fig. 3).

**Fig. 4:**
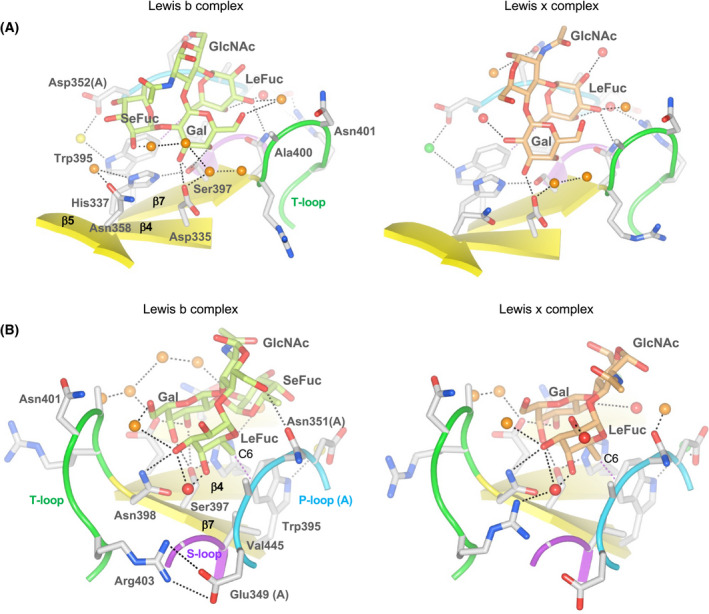
Details of HBGA binding to the P dimers. (A) The π–π stacking interaction formed between His337 and Trp395, and the residues responsible for Gal binding. (B) The residues responsible for LeFuc and GlcNAc binding, and a possible salt‐bridge between Arg403 and Glu349 in the Lewis b complex. ‘C6’ indicates the C‐6 methyl group of the LeFuc residue. The structures in (A) and (B) were viewed from the opposite sides.

The binding modes of both Lewis antigens to the Vancouver P dimer are quite similar to those described in the previous reports for GI.2 [12], GI.7 [13] and GI.8 [14] noroviruses. In both the Lewis x complex and Lewis b complex, the Gal and LeFuc moieties are mainly responsible for the binding to the P domain, as indicated by the formation of numbers of direct and water‐mediated hydrogen bonds (Fig. 3). Asn398 (side chain carboxamide) and Asn401 (main chain nitrogen) on the T‐loop as well as Glu349 (main chain carbonyl oxygen) and Asn351 (main chain nitrogen) on the P‐loop from another protomer are involved in the interaction with LeFuc (α1–3/4 fucose) (Fig. 4). It should be noted that, in the Vancouver P dimer, the π–π stacking interaction formed by Trp395 and His337 has an impact on the C‐6 methyl group of the LeFuc moiety via a hydrophobic interaction (Fig. 4A and Fig. S2). Together with Trp395, Val445 on the S‐loop, which is located at the bottom of the HBGA binding site, creates an environment that is sufficiently hydrophobic to accept the C‐6 methyl group of the LeFuc residue (Fig. 4B). Val445 in the GI.9 protein corresponds to Val442 in the GI.8 protein [14], which shares the same role in the LeFuc binding between the two residues (Fig. 2). In the Lewis x complex, an additional contribution of the side chain of Arg403 on the T‐loop to the hydrogen bonding with LeFuc is observed (Fig. 4B). In contrast, in the Lewis b complex the side chain of Arg403 does not always support LeFuc binding; rather, it is sometimes located closer to Glu349 on the P‐loop from another protomer (Fig. 4B, left panel), resulting in the formation of a salt‐bridge between these side chains. These results may suggest that the side chain of Arg403 is flexible. On the other hand, it seems unlikely that the residues corresponding to Asn401 and Arg403 on the T‐loop are involved in the LeFuc binding in other GI noroviruses [10, 11, 12, 13, 14].

The Gal moiety is supported by the side chains of Asp335 and His337 on the β sheet (β4) preceding the P‐loop as well as by the side chain of Ser397 at the end of β7 (Fig. 4A and Fig. S2). In addition, the side chain of Ala400 contributes to a hydrophobic interaction with the C‐6 methylene of the Gal moiety (Fig. 4A). The Asp335‐His337 doublet and Ser397 are conserved in all GI noroviruses (Fig. 2), suggesting their crucial role in carbohydrate binding. It is likely that the Gal binding site is strengthened by an antiparallel interaction between β4 and β7, in which the main chain atoms of His337 are hydrogen‐bonded with the main chain atoms of Ser397 (Fig. 4B).

In contrast, the SeFuc moiety of the Lewis b antigen interacts poorly with the P dimer. Only the side chain nitrogen of His337 is involved in the SeFuc binding (Fig. 4A). Similarly, the GlcNAc residue is not likely crucial for the interaction with the P dimer. It was observed that the side chain oxygen of Asn351 interacted with O‐6 of GlcNAc in the Lewis b complex, and that the main chain oxygen formed a water‐mediated interaction with O‐1 in the Lewis x complex (Fig. 4B).

## Discussion

The GI.9 strain bound to all Lewis antigens tested, including both secretor species (Lewis b and Lewis y) and nonsecretor species (Lewis a and Lewis x; Fig. 1B). Lewis b and Lewis a antigens have the type 1 linkage (Gal–β1–3–GlcNAc), while Lewis y and Lewis x antigens have the type 2 linkage (Gal–β1–4–GlcNAc; Fig. 1C). Although the avidity for Lewis y tetrasaccharide is relatively low, the Vancouver VLPs tightly bound to Lewis antigens irrespective of the type of linkage. On the other hand, they did not bind to any of the ABH blood group antigens, although a quite weak binding was observed for the H antigen (Fig. 1B), raising a possibility that GI.9 noroviruses infect humans independently of their blood types.

To understand the structural basis for Lewis antigen recognition, the crystals of the Vancouver P dimers were soaked with Lewis b tetrasaccharide or Lewis x trisaccharide. Both Lewis saccharides were accommodated in the crevasse formed by the T‐loop and S‐loop from one protomer and the P‐loop from another protomer, and these three loop structures played an exclusive role in the binding of LeFuc and Gal of Lewis antigens (Fig. 4). The HBGA binding interface on the Vancouver P dimer in addition to the mode of HBGA binding is similar to that on the P dimer of the GI.8 Boxer strain [14], which is also known as a Lewis binding species. In spite of the type of Gal–GlcNAc linkage, the amino acid residues interacting with the LeFuc and Gal moieties were almost equivalent in the Lewis b and Lewis x complexes. It should be noted that the π–π interaction formed by Trp395 and His337 affects the binding of the LeFuc (α1–3/4 fucose) moiety in the Vancouver P dimer; namely, the side chain of Trp395 points toward the C‐6 methyl of LeFuc (Fig. 4A). On the other hand, the corresponding His‐Trp interaction found in the other GI noroviruses supported a hydrophobic interaction with the SeFuc (α1–2 fucose) moiety rather than the LeFuc moiety [10, 11, 12, 13].

The GI.8 Boxer VP1 protein does not bind to the Lewis x antigen [14], while GI.9 VLPs appear to bind to this HBGA with high avidity (Fig. 1B). It is noteworthy that the side chain of Arg403 on the T‐loop participates in Lewis x binding via the water‐mediated interaction with the LeFuc moiety (Fig. 4B). Arg403 corresponds to Thr400 in GI.8 Boxer (Fig. 2), which does not contribute to Lewis antigen binding [14]. Thus, this difference might explain the reason for the difference in Lewis x binding ability between the two strains. In the Lewis b complex, the side chain of Arg403 moves outward so as to interact with Glu349 on the P‐loop from another protomer, which is also characteristic of GI.9. This might help to strengthen the integrity of the HBGA binding site. On the other hand, the GI.7 TCH‐060 P dimer binds to Lewis x, and the complex structure has been solved [13]. In this protein, amino acid residues on the T‐loop do not contribute to the interaction with LeFuc, but rather exclusively support the interaction with the Gal moiety, indicating the difference in architecture of the HBGA binding site.

The β1–3 galactose of Lewis b and the β1–4 galactose of Lewis x occupied a similar position in the respective complexes, and this was also the case for the LeFuc residue; that is, the α1–4 fucose of Lewis b and the α1–3 fucose of Lewis x also occupied similar positions (Fig. 4). In contrast, the α1–2 fucose residue (SeFuc) in the Lewis b tetrasaccharide was held by only a hydrogen bond with His337 (Fig. 4), reinforcing the idea that Lewis antigens were recognized by P domain proteins mainly via their LeFuc and Gal residues. This is consistent with the biochemical data showing that both Lewis a (type 1) and Lewis x (type 2) are recognized by VLPs to the same degree (Fig. 1B), indicating that the difference in the type of the Gal–GlcNAc linkage is not critical.

This raises a question: Why do the GI.9 VLPs favor Lewis b (type 1) over Lewis y (type 2)? Both antigens are composed of the same components: SeFuc, LeFuc, Gal, and GlcNAc, but the orientation of GlcNAc is completely different between them (Fig. 4). The crystal structures of the Lewis b‐bound and Lewis x‐bound P dimers indicate that the hydroxyl group at C‐1 of GlcNAc is exposed to a solvent. Considering that the LeFuc and Gal residues have almost the same placement in the HBGA binding site in almost the same manner in both complexes, SeFuc, LeFuc, and Gal of Lewis y tetrasaccharide would be situated in a similar way. In a study of the GI.8 strain [14], it was clearly shown that Lewis b and Lewis y were captured in an analogous fashion in the respective complexes, although more hydrogen bonds were involved in the binding of Lewis b than in the binding of Lewis y. This might be the case for the GI.9 P dimers. In addition, the dissociation of Lewis y might be more rapid than that of Lewis b in the GI.9 P dimers, considering that our HBGA binding assay is an endpoint assay that reflects the equilibrium between association and dissociation, and that there are several washing steps during a series of operations, which would allow a portion of the VLPs to be released from immobilized HBGAs.

In another case, we obtained seemingly contradictory results that the Vancouver VLPs did not bind to Lewis a trisaccharide‐BSA conjugates (data not shown), but that they did bind to the BSA conjugates of lacto‐*N*‐fucopentaose II, which included the Lewis a trisaccharide moiety in their terminals (Fig. 1C). This observation suggests that, in some saccharide conjugates, terminal oligosaccharides do not project outward to allow the binding of VLPs. The numbers of saccharide components and/or the length of the linker between saccharide and albumin might affect the binding property. Therefore, we cannot exclude the possibility that the configuration or orientation of Lewis y tetrasaccharide in the HSA conjugates used for the binding assay is not always optimal to function as a ligand for the GI.9 VLPs, although moderate Lewis y binding was observed (Fig. 1B). Thus, the HBGA binding assay may not have precisely revealed all of the characteristics of VLPs; if so, this would be a notable limitation of this study.

Another question is why the GI.9 Vancouver strain does not bind to the H antigen. The H antigen has the SeFuc residue at the terminus as well as the Lewis b/y antigens (Fig. 1C). As described above, the Vancouver P dimer mainly recognizes Gal and LeFuc residues of Lewis antigens via a number of hydrogen bonds, but the interaction with SeFuc is rather poor. This indicates that the presence of LeFuc is the major determinant for selection of HBGAs by the GI.9 noroviruses. Thus, it is likely insufficient to bind the H antigen having only the SeFuc moiety. It is interesting that, in the GI.7 TCH‐060 strain, SeFuc of the Lewis y antigen is held by the side chain of Arg351 on β5, which was fixed by the interaction with Asp353, as well as by a hydrophobic interaction between Trp385 and the C‐6 methyl of SeFuc [13]. Arg351 in the GI.7 strain also contributes to the binding of the H antigen and the A antigen; the side chain of Arg351 interacts with SeFuc in the H antigen complex, and with the acetamide of the terminal GalNAc residue in the A antigen complex [13]. In addition, Gln342 and Asp344 on β5 in the GI.1 strain [10] and Ser352 in the GI.2 strain [12] are involved in the interaction with SeFuc of the H antigen. On the other hand, in the GI.9 Vancouver strain, there is no contribution of amino acid residues on β5 for the interaction with SeFuc of the Lewis b antigen; moreover, Trp395, which corresponds to Trp385 of the GI.7 strain, supports the binding of LeFuc, as described above. Also in the GI.8 Boxer, another strain that binds eagerly to the Lewis antigens, residues on β5 do not contribute to the binding of HBGA [14].

Neither the GI.9 Vancouver VLPs nor the GI.8 Boxer VLPs [14, 35] bind to the A and B antigens (Fig. 1B). This might be because the SeFuc binding site is degenerated in both VLPs, as described for GI.8 [14]. In the GI.1 [10], GI.2 [12] and GI.7 [13] strains, the *N*‐acetyl group of the terminal GalNAc residue of the A antigen occupied the SeFuc binding site, and the SeFuc residue of the A antigen was located on the opposite side near the T‐loop, which was distinct from the case for the H antigen binding in the respective strains. As shown in Fig. 2, the T‐loops of the GI.1 [10] and GI.2 [12] strains are much longer than that of the GI.9 Vancouver strain, and it might therefore be possible that the T‐loop adopts a bent and open conformation, creating a space sufficiently large to accept the SeFuc residue of the A antigen. On the other hand, the GI.9 and GI.8 [14] strains have the shortest T‐loops among the GI strains, and it may therefore be unlikely that amino acid residues on the T‐loop could form the interaction with SeFuc of the A antigen.

Although there is some variation in the binding profiles, binding to Lewis antigens is likely a common characteristic of the GI noroviruses [37], suggesting that Lewis antigen(s) play a critical role in infection by the GI noroviruses. Although the structural analyses revealed that HBGA binding did not induce gross conformational change of the P domain [10, 11, 12, 13, 14], it is still unknown whether this is the case for VLPs and infectious virus particles. Therefore, we should not rule out the possibility that HBGA is a principal receptor for infection, since it actually affects the susceptibility to some genotypes of noroviruses [23, 24]. Investigating this possibility should be a matter of priority for understanding the first step of the norovirus life cycle.

## Conflict of interest

The authors declare no conflicts of interest.

## Author contributions

TK‐S, MS, and YS conceived and supervised the study; MK‐M, KK, KH, and YS performed the experiments; MK‐M, KK, NS, and KM analyzed the protein structures; TK‐S and YS wrote the article; all members commented on the article.

## Supporting information


**Fig. S1**. Crystal structure of the GI.9 Vancouver P dimer in apo form.
**Fig. S2**. Interaction map of the Lewis antigen binding site in the Vancouver P dimer.

## Data Availability

The structural data obtained in this study were deposited in the Protein Data Bank (PDB) under the accession codes 7VP0 (apo‐protein), 7VS8 (Lewis b complex), and 7VS9 (Lewis x complex).

## References

[1] Robilotti E , Deresinski S , Pinsky BA . Norovirus. Clin Microbiol Rev. 2015;28:134–64. 10.1128/CMR.00075-14. 25567225 PMC4284304

[2] Chhabra P , de Graaf M , Parra GI , Chan MCW , Green K , Martella V , et al. Updated classification of norovirus genogroups and genotypes. J Gen Virol. 2019;100:1393–406. 10.1099/jgv.0.001318. 31483239 PMC7011714

[3] Lopman B . Global burden of norovirus and prospects for vaccine development. CDC; 2015. [cited 2015 Aug]. Available from https://www.cdc.gov/norovirus/downloads/global‐burden‐report.pdf

[4] Prasad BV , Hardy ME , Dokland T , Bella J , Rossmann MG , Estes MK . X‐ray crystallographic structure of the Norwalk virus capsid. Science. 1999;286:287–90. 10514371 10.1126/science.286.5438.287

[5] Conley MJ , McElwee M , Azmi L , Gabrielsen M , Byron O , Goodfellow IG , et al. Calicivirus VP2 forms a portal‐like assembly following receptor engagement. Nature. 2019;565:377–81. 10.1038/s41586-018-0852-1. 30626974

[6] Jiang X , Wang M , Graham DY , Estes MK . Expression, self‐assembly, and antigenicity of the Norwalk virus capsid protein. J Virol. 1992;66:6527–32. 1328679 10.1128/jvi.66.11.6527-6532.1992 PMC240146

[7] White LJ , Hardy ME , Estes MK . Biochemical characterization of a smaller form of recombinant Norwalk virus capsids assembled in insect cells. J Virol. 1997;71:8066–72. 9311906 10.1128/jvi.71.10.8066-8072.1997 PMC192173

[8] Someya Y , Shirato H , Hasegawa K , Kumasaka T , Takeda N . Assembly of homogeneous norovirus‐like particles accomplished by amino acid substitution. J Gen Virol. 2011;92:2320–3. 10.1099/vir.0.033985-0. 21715601

[9] Hasegawa K , Someya Y , Shigematsu H , Kimura‐Someya T , Kumasaka T . Crystallization and preliminary X‐ray analysis of 23‐nm virus‐like particles from norovirus Chiba strain. Acta Crystallogr F Struct Biol Commun. 2017;73(Pt 10):568–73. 10.1107/S2053230X17013759. 28994405 PMC5633924

[10] Choi JM , Hutson AM , Estes MK , Prasad BV . Atomic resolution structural characterization of recognition of histo‐blood group antigens by Norwalk virus. Proc Natl Acad Sci USA. 2008;105:9175–80. 10.1073/pnas.0803275105. 18599458 PMC2453692

[11] Bu W , Mamedova A , Tan M , Xia M , Jiang X , Hegde RS . Structural basis for the receptor binding specificity of norwalk virus. J Virol. 2008;82:5340–7. 10.1128/JVI.00135-08. 18385236 PMC2395213

[12] Kubota T , Kumagai A , Ito H , Furukawa S , Someya Y , Takeda N , et al. Structural basis for the recognition of Lewis antigens by genogroup I norovirus. J Virol. 2012;86:11138–50. 10.1128/JVI.00278-12. 22855491 PMC3457155

[13] Shanker S , Czako R , Sankaran B , Atmar RL , Estes MK , Prasad BV . Structural analysis of determinants of histo‐blood group antigen binding specificity in genogroup I noroviruses. J Virol. 2014;88:6168–80. 10.1128/JVI.00201-14. 24648450 PMC4093872

[14] Hao N , Chen Y , Xia M , Tan M , Liu W , Guan X , et al. Crystal structures of GI.8 Boxer virus P dimers in complex with HBGAs, a novel evolutionary path selected by the Lewis epitope. Protein Cell. 2015;6:101–16. 10.1007/s13238-014-0126-0. 25547362 PMC4312760

[15] Tan M , Xia M , Chen Y , Bu W , Hegde RS , Meller J , et al. Conservation of carbohydrate binding interfaces – evidence of human HBGA selection in norovirus evolution. PLoS One. 2009;4:e5058. 10.1371/journal.pone.0005058. 19337380 PMC2660415

[16] Tan M , Jiang X . Norovirus gastroenteritis, carbohydrate receptors, and animal models. PLoS Pathog. 2010;6:e1000983. 10.1371/journal.poat.1000983. 20865168 PMC2928792

[17] Tan M , Jiang X . Norovirus‐host interaction: multi‐selections by human histo‐blood group antigens. Trends Microbiol. 2011;19:382–8. 10.1016/j.tim.2011.05.007. 21705222 PMC3149758

[18] Tan M , Jiang X . Histo‐blood group antigens: a common niche for norovirus and rotavirus. Expert Rev Mol Med. 2014;16:e5. 10.1017/erm.2014.2. 24606759 PMC12406300

[19] Liu W , Chen Y , Jiang X , Xia M , Yang Y , Tan M , et al. A Unique human norovirus lineage with a distinct HBGA binding interface. PLoS One. 2015;11:e1005025. 10.1371/journal.pone.1005025. PMC4493018 26147716

[20] Singh BK , Leuthold MM , Hansman GS . Human noroviruses’ fondness for histo‐blood group antigens. J Virol. 2015;89:2024–40. 10.1128/JVI.02968-14. 25428879 PMC4338890

[21] Singh BK , Leuthold MM , Hansman GS . Constraints on human norovirus binding to histo‐blood group antigens. mSphere. 2016;1:e00049‐16. doi: 10.1128/mSphere.00049-16. 27303720 PMC4894678

[22] Koromyslova A , Tripathi S , Morozov V , Schroten H , Hansman GS . Human norovirus inhibition by a human milk oligosaccharide. Virology. 2017;508:81–9. 10.1016/j.virol.2017.04.032. 28505592

[23] Nordgren J , Svensson L . Genetic susceptibility to human norovirus infection: an update. Viruses. 2019;11:E226. 10.3390/v11030226. 30845670 PMC6466115

[24] Nordgren J , Kindberg E , Lindgren PE , Matussek A , Svensson L . Norovirus gastroenteritis outbreak with a secretor‐independent susceptibility pattern, Sweden. Emerg Infect Dis. 2010;16:81–7. 10.3201/eid1601.090633. 20031047 PMC2874438

[25] Yabuki T , Motoda Y , Hanada K , Nunokawa E , Saito M , Seki E , et al. A robust two‐step PCR method of template DNA production for high‐throughput cell‐free protein synthesis. J Struct Funct Genomics. 2007;8:173–91. 10.1007/s10969-007-9038-z. 18167031

[26] Katsura K , Matsuda T , Tomabechi Y , Yonemochi M , Hanada K , Ohsawa N , et al. A reproducible and scalable procedure for preparing bacterial extracts for cell‐free protein synthesis. J Biochem. 2017;162:357–69. 10.1093/jb/mvx039. 28992119 PMC7109869

[27] Ueno G , Kanda H , Hirose R , Ida K , Kumasaka T , Yamamoto M . RIKEN structural genomics beamlines at the SPring‐8; high throughput protein crystallography with automated beamline operation. J Struct Funct Genomics. 2006;7:15–22. 10.1007/s10969-005-9005-5. 16645781

[28] Ito S , Ueno G , Yamamoto M . DeepCentering: fully automated crystal centering using deep learning for macromolecular crystallography. J Synchrotron Rad. 2019;26(Pt 4):1361–6. 10.1107/S160057751900434X. PMC6613109 31274465

[29] Okazaki N , Hasegawa K , Ueno G , Murakami H , Kumasaka T , Yamamoto M . Mail‐in data collection at SPring‐8 protein crystallography beamlines. J Synchrotron Rad. 2008;15(Pt 3):288–91. 10.1107/S0909049507064679. PMC2394786 18421161

[30] Murakami H , Ueno G , Shimizu N , Kumasaka T , Yamamoto M . Upgrade of automated sample exchanger SPACE. J Appl Cryst. 2012;45:234–8. 10.1107/S0021889812003585.

[31] Kabsch W . XDS. Acta Crystallogr D Biol Crystallogr. 2010;66(Pt 2):125–32. 10.1107/S0907444909047337. 20124692 PMC2815665

[32] Otwinowski Z , Minor W . Processing of X‐ray diffraction data collected in oscillation mode. Methods Enzymol. 1997;276:307–26. 10.1016/S0076-6879(97)76066-X. 27754618

[33] McCoy AJ , Grosse‐Kunstleve RW , Adams PD , Winn MD , Storoni LC , Read RJ . Phaser crystallographic software. J Appl Crystallogr. 2007;40(Pt 4):658–74. 10.1107/S0021889807021206. 19461840 PMC2483472

[34] Adams PD , Afonine PV , Bunkóczi G , Chen VB , Davis IW , Echols N , et al. PHENIX: a comprehensive Python‐based system for macromolecular structure solution. Acta Crystallogr D Biol Crystallogr. 2010;66(Pt 2):213–21. 10.1107/S0907444909052925. 20124702 PMC2815670

[35] Emsley P , Cowtan K . Coot: model‐building tools for molecular graphics. Acta Crystallogr D Biol Crystallogr. 2004;60(Pt 12):2126–32. 10.1107/S0907444904019158. 15572765

[36] Pettersen EF , Goddard TD , Huang CC , Couch GS , Greenblatt DM , Meng EC , et al. UCSF Chimera–a visualization system for exploratory research and analysis. J Comput Chem. 2004;25:1605–12. 10.1002/jcc.20084. 15264254

[37] Huang P , Farkas T , Zhong W , Tan M , Thornton S , Morrow AL , et al. Norovirus and histo‐blood group antigens: demonstration of a wide spectrum of strain specificities and classification of two major binding groups among multiple binding patterns. J Virol. 2005;79:6714–22. 10.1128/JVI.79.11.6714-6722. 15890909 PMC1112114

