# Supplementary material for: Lewis fucose is a key moiety for the recognition of histo‐blood group antigens by GI.9 norovirus, as revealed by structural analysis
Source: FEBS Open Bio. 2022 Jan 30;12(3):560–70. doi: 10.1002/2211-5463.13370 (PMC8886331; doi:10.1002/2211-5463.13370)
Supplement: Supplementary file 1 — Fig. S1. Crystal structure of the GI.9 Vancouver P dimer in apo form. Fig. S2. Interaction map of the Lewis antigen binding site in the Vancouver P dimer. [file FEB4-12-560-s001.pdf]

(A)

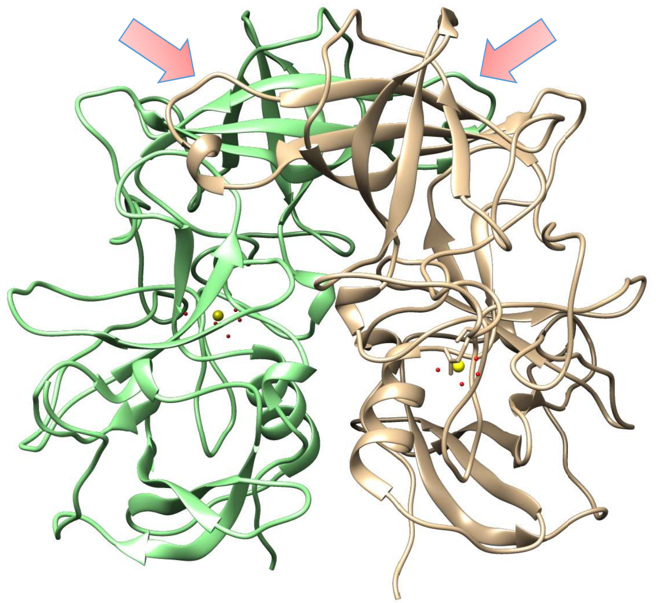

(B)

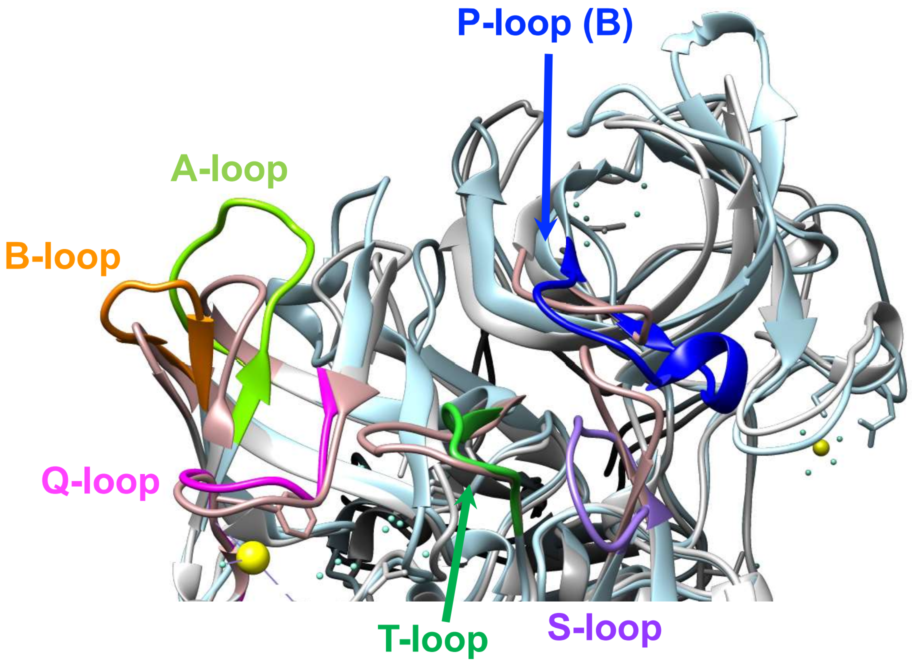

Supplementary figure 1. Crystal structure of the GI.9 Vancouver P dimer in apo form. (A) Overall structure of the P dimer. *Red arrows* indicate the HBGA binding sites. (B) Comparison of the HBGA binding site between the Vancouver P dimer (*pale blue*) and the GI.1 Norwalk P dimer (*grey*) (PDB 2ZL5). These two structures are overlaid. Loop structures of the Vancouver P dimer are indicated in colors as shown in Figure 2. The corresponding loop structures of the Norwalk counterpart are indicated in *pale red*.

Lewis b complex

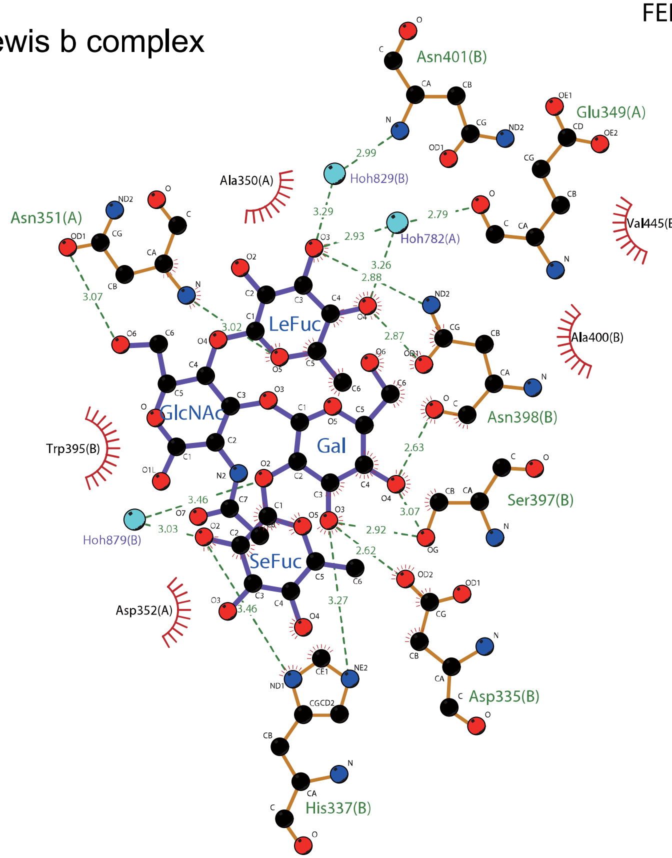

Lewis x complex

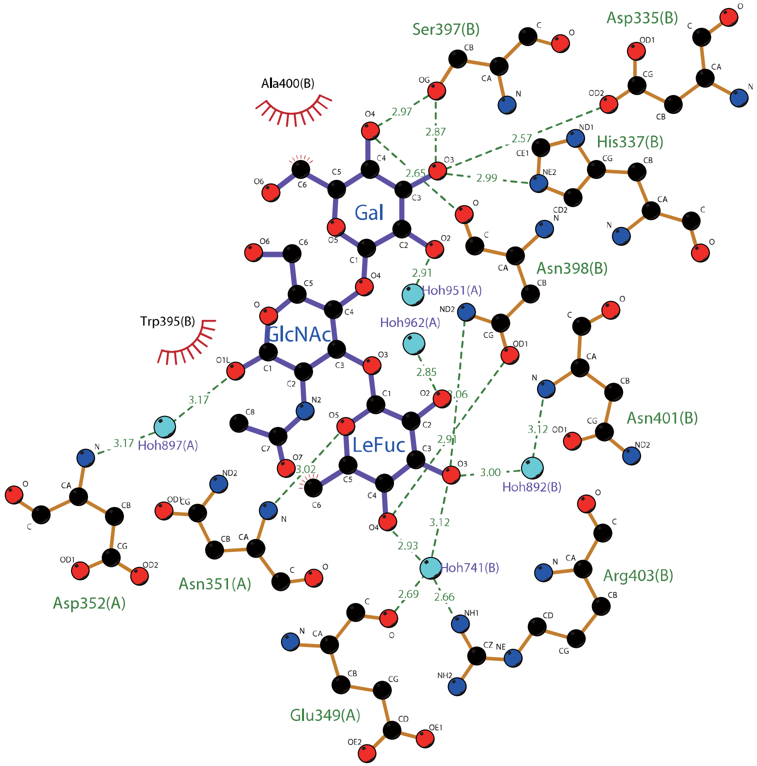

Supplementary figure 2. Interaction map of the Lewis antigen binding site in the Vancouver P dimer. Several amino acid residues and water molecules involved in the interaction with Lewis b and Lewis x antigens are depicted. “*(A)*” and “*(B)*” following amino acid and saccharide residues indicate the A and B subunit of the P dimer, respectively.
